# A dynamic capabilities view of improvement capability

**DOI:** 10.1108/JHOM-11-2018-0342

**Published:** 2019-11-07

**Authors:** Joy Furnival, Ruth Boaden, Kieran Walshe

**Affiliations:** Alliance Manchester Business School, The University of Manchester, Manchester, UK

**Keywords:** Dynamic capabilities, Orchestration, Microfoundations, Improvement capability

## Abstract

**Purpose:**

Organisations within healthcare increasingly operate in rapidly changing environments and present wide variation in performance. It can be argued that this variation is influenced by the capability of an organisation to improve: its improvement capability. However, there is little theoretical research on improvement capability. The purpose of this paper is to set out the current diverse body of research on improvement capability and develop a theoretically informed conceptual framework.

**Design/methodology/approach:**

This paper conceptualises improvement capability as a dynamic capability. This suggests that improvement capability is comprised of organisational routines that are bundled together, and adapt and react to organisational circumstances. Existing research conceptualises these bundles as three elements (microfoundations): sensing, seizing and reconfiguring. This conceptualisation is used to explore how improvement capability can be understood, by inductively categorising eight dimensions of improvement capability to develop a theoretically informed conceptual framework.

**Findings:**

This paper shows that the three microfoundations which make up a dynamic capability are present in the identified improvement capability dimensions. This theoretically based conceptual framework provides a rich explanation of how improvement capability can be configured.

**Originality/value:**

Identifying the component parts of improvement capability helps to explain why some organisations are less successful in improvement than others. This theoretically informed framework can support managers and policy makers to identify improvement capability dimensions in need of development. Further empirical research, particularly in non-market settings, such as publicly funded healthcare is required to enhance understanding of improvement capability and its configuration.

## Introduction

Large, persistent and unexplained variations in performance persist in healthcare. This variation cannot all be explained by differences in the health status of populations, geographical differences or by better outcomes in some higher spending regions (Lavergne *et al.*, 2016). This suggests that policy reform aimed at system wide quality and efficiency may prove promising (Lavergne *et al.*, 2016) in reducing variation. Therefore, organisations continue to seek better ways to improve performance, including a focus on the quality and safety of healthcare. Talbot (2010) describes four approaches that can be taken to ensure improved performance: managerial and contractual interventions; market mechanisms set through the policy context such as regulation; user choice and voice; and interventions to develop improvement capability. Improvement capability can be defined as “the organisational ability to intentionally and systematically use improvement approaches, methods and practices, to change processes and products/services to generate improved performance” (Furnival *et al.*, 2017, p. 606). However, there has been little theoretically informed research on how to develop improvement capability (Talbot, 2010; Downe *et al.*, 2010; Dixon-Woods and Martin, 2016). This deficit is addressed in this paper.

Developing theoretically informed research on improvement capability is important as there remains a lack of knowledge about what improvement approaches, and methods are effective and in what contexts (Dixon-Woods and Martin, 2016). In addition, whilst the term improvement capability is a widely used term and is seen as a useful concept, it has considerable ambiguity with a lack of consensus about what constitutes improvement capability and how it is operationalised.

The paper is organised as follows: first, the methodological approach to the paper is detailed; then the concept of improvement capability is introduced, followed by a description of three theories of performance improvement, including the dynamic capabilities view (Helfat *et al.*, 2007). The current diverse body of research regarding improvement capability is used to develop a theoretically based conceptual framework using the dynamic capabilities view (Helfat *et al.*, 2007). The framework highlights and explains the elements of improvement capability that need to be considered in practice. What is missing in the literature is a conceptual framework to take account of the inter-relationships between micro, meso and macro factors implicated in organisational improvement, to guide practical service improvement and improvement capability itself. The article contributes to the literature by identifying and organising the dimensions of improvement capability, drawing on the microfoundations of dynamic capabilities (Barney and Felin, 2013).

## Methodological approach

The theoretical framework developed in this paper is the second element of a larger scale research study. It was developed by drawing on a synthesis of existing improvement capability frameworks and assessment instruments detailed in the integrative literature review (Furnival *et al.*, 2017) which was conducted in phase 1 of the larger study. The methodological approach to this integrative literature review started with a general literature review, to understand what is already known about improvement capability and relevant theoretical perspectives. The approach to the review drew initially on the seminal text from Bessant and Francis (1999). Next, backwards and forwards citation searching was used to inform a wider reading of the literature. Literature was identified using several search keyword strategies, including thesaurus terms, free-text terms and broad-based terms to optimise searching for qualitative evidence (Shaw *et al.*, 2004). The search was not limited by sector or academic discipline to ensure the widest possible field. Multiple cycles of iterative literature searching and browsing took place to support serendipitous discovery (Greenhalgh and Peacock, 2005) including the use of snowballing strategies and the use of personal knowledge and connections to seek out more obscure literature until saturation was reached (Randolph, 2009; Jones *et al.*, 2019).

Frameworks can be basic, comprehensive, theory driven, causal or descriptive (Miles and Huberman, 1994). The process of developing the conceptual framework within this article involved building theory through the identification of the most pertinent constructs from the integrative literature review (Furnival *et al.*, 2017) and then using visual processes of model development. Visual processes of model development in NVivo10 were used to initially track the constructs as nodes. The functionality within NVivo10 was used to move and consider different relationships between the constructs and the model, and these were discussed until consensus was reached. This was performed as an iterative cyclical process between the two elements of the research study, to inductively develop refinements to the framework over time and as data were collected.

## Improvement capability

The integrative literature review (Furnival *et al.*, 2017) revealed that the concept is broad, ambiguous and fragmented, with high levels of terminological heterogeneity across many disciplines. Different definitions identified from the literature (Furnival *et al.*, 2017) are presented in Table I. One perspective describes improvement capability as an organisational wide process of focussed and sustained incremental innovation to maximise creative problem solving (Bessant and Francis, 1999; Bessant and Caffyn, 1997). Whereas, Adler *et al.* (2003) describe improvement capability as supporting innovation across an organisation. Alternatively, Peng *et al.* (2008) distinguish improvement capability as focussing on existing products and processes, rather than innovation capability. Interestingly, later definitions from healthcare take a human capital and actor driven perspective, where improvement is delivered through individuals and their leadership. This perspective describes improvement capability as encouraging staff to learn skills for conducting improvements (Kaminski *et al.*, 2014; Bevan, 2010). However, Babich *et al.* (2016) return to the earlier definition provided by Adler *et al.* (2003), which takes a process view indicating that organisational improvement is generated through following particular procedures and methods. Recognising this heterogeneity in the conceptualisation of improvement capability, it is important that the literature consolidates and builds on previous research in a structured way (Barreto, 2010). Therefore, this paper takes a process view of improvement capability and uses the definition of improvement capability synthesised in Furnival *et al.* (2017) drawing from the wide-ranging literature as a follow-on and related study. This paper extends this perspective further by using theory to explore improvement capability.

The integrative literature review of frameworks used to assess improvement capability details 70 instruments and identified more than 22 heterogeneous constructs (Furnival *et al.*, 2017). The review synthesises these constructs into eight dimensions of improvement capability, and these are described in Table II. The dimensions can be viewed as eight high level routines that bundle to form improvement capability. However, further theorisation of the improvement capability dimensions is required, as this review only assembles existing literature on improvement capability into themes. It does not indicate how or why each dimension is important or whether one dimension may be more important than another, or must be in place in advance of other dimensions. In addition, research has indicated that improvement approaches used to develop capability have been criticised as insufficiently theoretical and explanatory research to explain why and how improvement “works” and where not has largely fallen behind increased use of improvement approaches (Dixon-Woods and Martin, 2016; Young and McClean, 2008).

To summarise, improvement capability is a widely used term with considerable ambiguity regarding its definition, meaning and operationalisation. Nevertheless, the integrative literature review (Furnival *et al.*, 2017) indicated that the concept of improvement capability is under-theorised and would benefit from further theoretical development. The next section considers the theories concerned with organisational capability and how they can provide theoretical foundations.

## Theoretical perspectives

There are many different theories that could be used to underpin research concerning improvement capability, and Table III outlines three capability led theories of performance drawing from Talbot (2010). These are the resource-based view (Barney, 1991), organisational ambidexterity (O’Reilly and Tushman, 2004) and the dynamic capabilities view (Helfat *et al.*, 2007). These widely accepted capability led theories of performance have had a significant impact on debates and are interdisciplinary in nature. Table III highlights the main principles, focus and components of each theory, and provides information on the support for and the critiques of these theories. Each are now described in turn.

First, the resource-based view proposes that persistent superior performance is related to organisational strategic resources. It claims that organisations are comprised of a mix of tangible and intangible resources, such as physical, human and organisational capital (Barney, 1991). Differences in the distribution of these resources within organisations are understood to account for performance variation. The resource-based view suggests that internal forces drive firms’ decision making on how best to deploy organisational resources (Rumelt, 1982; Wernerfelt, 1984). Strategic resources must be valuable, rare, inimitable and exploitable (known as the VRIN framework) (Barney, 1991; 1995).

The resource-based view focusses attention on the internal resources or strengths within an organisation to manage uncertainty, rather than capitalising on the opportunities presented by a changing external environment. Addressing these opportunities depends on the scope to invest in new resources. Examples include patents, reputation and unique knowledge. The resource-based view is criticised for being static and accounting insufficiently for changes in the external environment of organisations (Teece, 2012; Helfat *et al.*, 2007).

Second, organisational ambidexterity claims that to improve performance, organisations need to both simultaneously pursue both explorative (discontinuous) and exploitative (incremental) innovation (O’Reilly and Tushman, 2004). This is the notion of balancing the need to deliver both incremental and radical change simultaneously within an organisation to secure survival and long-term performance. Exploration is described as a focus on organisation discovery, autonomy, learning new things and innovation. Exploitation is described as a focus on efficiency, control, certainty and variation reduction (March, 1991), in other words, putting existing knowledge into practice. Gibson and Birkinshaw (2004) indicate that there are always trade-offs to be made between exploitation and exploration. Whilst these trade-offs can never entirely be eliminated, the most successful organisations reconcile them to a larger degree, and in so doing enhance their long-term performance.

Third, the dynamic capabilities view claims that superior and sustained performance depends on the organisational capacity to purposefully create, extend and modify its resource base through bundles of organisational routines, which must be sustained over time (Su *et al.*, 2014; Ambrosini and Bowman, 2009; Helfat *et al.*, 2007). Capabilities and routines differ across and between organisations, as they have different levels of resources or may have created routines differently (Hitt, Carnes and Xu, 2016). For example, the simple and structured improvement and leadership routines used widely throughout the Toyota Motor Corporation have been difficult to copy in other organisations (Rother, 2009). It is argued that organisations with more dynamic capabilities will outperform organisations with less, and that dynamic capabilities both mediate and underpin high performance (Felin *et al.*, 2015). This is said to particularly be the case in dynamic environments where there is ongoing uncertainty through changing technology, competition or political mandates, and organisations need to adapt to these changing conditions (Felin *et al.*, 2015). The orchestration and configuration of the organisational routines necessary to enact a dynamic capability (Sirmon *et al.*, 2007) may be useful in explaining why some organisations are less successful in performance improvement than others. Orchestration is required to ensure that excessive focus on some organisational routines does not lead to an imbalance or is at the expense of another, sub-optimising the development of the bundle of routines within the dynamic capability.

Whilst each theory has emerged from different streams of research, there is considerable theoretical and empirical overlap and similarity in the levels of evidence. Vogel and Güttel (2013) confirm the findings from Di Stefano *et al.* (2010) that there are similar theoretical foundations for the dynamic capabilities view, the resource-based view and organisational ambidexterity within management research. These three theories were developed within the private sector, mostly within rapidly changing environments where innovation has been seen as essential to ensure competitive advantage and performance, such as semi-conductor processing and electronics (Easterby-Smith *et al.*, 2009).

The three theories are critiqued similarly in four main areas:

1. There is insufficient conceptualisation, and vague and inconsistent definitions are used (Arend and Bromiley, 2009; Kraaijenbrink *et al.*, 2010; Birkinshaw and Gupta, 2013; Vogel and Güttel, 2013).
2. The theories place a focus and emphasis on macro perspectives rather than microelements and their assembly (Molina-Azorín, 2014; Turner *et al.*, 2013; Felin *et al.*, 2015). The emphasis on macro perspectives means that the details of what makes up capability led interventions and how they may be measured and developed is overlooked.
3. The theories have inadequate measurement and prediction which hinders the development of empirical evidence (Hinterhuber, 2013; Junni *et al.*, 2013; Pavlou and El Sawy, 2011).
4. There is a lack of research into understanding the role of managers and their role in decision making (Turner *et al.*, 2013; Sirmon *et al.*, 2007; Eggers and Kaplan, 2013).

There is considerable overlap across the three capability led theories of performance described in this paper, in content and critique, and there is unclear differentiation between these related theories (Hitt, Xu and Carnes, 2016). However, there are some distinctive differences between the theories. In particular, the dynamic capabilities view has a focus on how routines bundle, orchestrate and interact and it is the view that appears to be used in the most substantial body of literature. Further, organisational ambidexterity has itself been described as a dynamic capability (Helfat and Winter, 2011; O’Reilly and Tushman, 2008), as has improvement capability (Bessant and Francis, 1999). In addition, the dynamic capabilities view takes a process view of performance and recognises the interplay between internal and external environments of organisations and recognises that capabilities can continually develop, bundle, reform, interact and atrophy over time in response to or anticipation of these contextual changes. Finally, the dynamic capabilities view has rarely been applied or developed within a healthcare and public sector context. There has been little research using any capability led resource-based theories of performance within the public sector (Burton and Rycroft-Malone, 2014; Easterby-Smith *et al.*, 2009; Smith and Umans, 2015). Therefore, the dynamic capabilities view is used in this paper to understand how improvement capability dimensions are configured and to contribute further to the understanding of the dynamic capabilities view within a healthcare context.

## A dynamic capabilities view of improvement capability

This section considers how the dynamic capabilities view contributes to the understanding of improvement capability, using the concept of microfoundations. Dynamic capabilities build from three constituent parts or “microfoundations”: sensing, seizing and reconfiguring (Helfat and Martin, 2014; Teece, 2012). Microfoundations have been described as a way of unpacking ambiguous concepts to examine their origins and evolution as a function of lower level factors (Barney and Felin, 2013). Using microfoundations as an analytical approach allows collective concepts to be unpacked into individual factors to understand their impact (Barney and Felin, 2013). Barney and Felin (2013) describe microfoundations as a pragmatic and important approach for examining collective constructs. This is important given the conceptual inconsistency identified from the integrative literature review (Furnival *et al.*, 2017) and the need for operational definitions to support development plans, measurement and evaluation.

Using the view of dynamic capabilities as comprising microfoundations, the dimensions of improvement capability identified in the integrative literature review (Furnival *et al.*, 2017) are studied (Table IV). The descriptions for each improvement capability dimension found in the integrative literature review (Furnival *et al.*, 2017) are compared with each microfoundation and inductively categorised as described in the methodological approach to ascertain if the three microfoundations of dynamic capability are present. Giudici and Reinmoeller (2012) state that dynamic capabilities research needs to strive for clarity in definitions in order to prevent reification and to build incremental knowledge. Table IV shows that the improvement capability dimensions can be viewed as microfoundations, which improves the clarity of definition using a theoretical analysis.

### Sensing

The microfoundation of sensing is described as the skills and processes to detect and process emerging opportunities before they fully materialise (Denrell *et al.*, 2003). For example, an example of sensing microfoundation activities would be a research and development process. Sensing focusses on the organisation identifying ideas and changes within the ecosystem that require responses. This includes viewpoints, opinions, technological advances and external requirements, such as regulations that change continuously and ongoing developments from stakeholders, suppliers as well as changing service-user requirements. Stakeholders, suppliers and service users tend to be in roles external to an organisation yet are still members of the wider system. Such roles are not directly decision-making roles, nor directly able to influence change or reconfiguration actions. Two improvement capability dimensions of service-user focus and stakeholder and supplier focus were categorised as “sensing” microfoundations. This group of dimensions is significant within a health or public sector setting, since ensuring that patient and taxpayer concerns, expectations, priorities, issues, technological developments and other changes by suppliers are considered when considering improvements to be enacted is important. Considering this microfoundation will ensure that views of the wider health and care system are taken into account.

### Seizing

Seizing is the microfoundation that encompasses routines and processes that ensure strategic choices and associated investments are made in these emerging opportunities within organisations (Helfat and Martin, 2014; Teece, 2012). This includes the decisions to make health service or system improvements, such as decisions that inform service-user complaints, suggestions or staff ideas to take forwards. Commitment to a particular strategic thrust by both the leadership and employees of an organisation is critical, and commitment is considered the prerequisite for sustained performance (Schreyögg and Kliesch-Eberl, 2007). The three dimensions of organisational culture, leadership commitment and employee commitment, were categorised as “seizing” microfoundations. These three dimensions relate to the internal environment of an organisation and the decision-making processes within to (dis)assemble capabilities in response and anticipation of opportunities identified, rather than the routines and actions that might directly lead to change and reconfiguration. The seizing microfoundation is significant within a health or public sector setting because there are many different and competing priorities from which leaders and teams may choose to improve, some of which may be clear through feedback or collation of other suggestions, but some may be hidden. This microfoundation ensures teams and staff feel able to suggest ideas and highlight issues, understand that they will be reviewed seriously and fairly, and that feedback will be shared openly if an idea is not progressed further. In addition, this microfoundation indicates that it is the role of all team members to contribute to improving, and seizing opportunities, rather than the role of a few in senior positions or important roles.

### Reconfiguration

Finally, the reconfiguring microfoundation encompasses organisational routines that enact and execute the decisions made by adapting established processes or acquiring new ones (Helfat and Martin, 2014; Teece, 2012). This includes implementing new processes and policies, whilst incrementally making changes to existing processes and policies. This encompasses methods and processes used to generate and monitor improvement activities, including the use of improvement approaches such as lean, together with the use of measurement systems and practices to develop and review plans and strategies. The three dimensions of process improvement and learning, data and performance, and strategy and governance, are directly related to action taking and processes to confirm and assure that action has been taken within an organisation. Therefore, these two dimensions were categorised as the “reconfiguring” microfoundations. The reconfiguring microfoundation is significant within a health or public sector setting as this microfoundation relates to the group that enact the change, and ensures it happens using data, methods and review processes.

### Inter-relationships

This categorisation process illustrates that the three microfoundations that make up a dynamic capability are present in the improvement capability dimensions identified in the improvement capability framework developed from the integrative literature review (Furnival *et al.*, 2017). For example, many healthcare organisations provide training for the use of improvement tools and techniques, and this could be categorised within the process improvement and learning dimension, as part of the reconfiguration microfoundation. However, the service-user focus dimension is needed to inter-relate with the process improvement and learning dimension to ensure that service-user requirements inform the improvement training, given that most improvement approaches emphasise the role of the service user (or customer) (Boaden and Furnival, 2016). An organisation without inter-relationships between dimensions categorised within the sensing and reconfiguration microfoundations may be ineffective at solving service user or customer needs. This could potentially lead to a “cosmetic” organisation; actively sensing and seizing new ideas and knowledge, and developing plans for their use, but unable to put its plans into practice and execute the changes required. This could also lead to an organisation giving a false impression and a sense of assurance to external agencies, service users and stakeholders, and leading to a delay in the reconfiguration of routines and persistent excessive variation in performance. For example, healthcare organisations who have used their data, such as service user and patient feedback, to identify an area where they have some performance challenges and actively sought out organisations that seem to be able to run a particular area well, but do not then enact changes. If all microfoundations are used, ideas can be exchanged between other organisations thereby identifying new ideas for improvement and reduced excessive variation in performance can be sought out. Areas that seem to offer the most benefit can be seized and then acted upon, reconfiguring services to see if performance improves. The organisation is then orchestrating and using all three microfoundations of improvement capability together as a bundle with the aim of eliminating the excessive variation in performance.

Without sensing, an organisation may appear arrogant, insular and fortress-like, seemingly unwilling to seek ideas and knowledge from outside the organisation and focussed on internally generated ideas for improvement based on an incomplete view of the opportunities and threats facing that organisation. This is where an organisation is actively seizing and developing improvement plans, strategies and measuring their outputs, but the improvements fail to meet external stakeholder, supplier and service-user needs. For example, a focus on improving product or service quality when the external stakeholders, customers and service users want improvements such as lower priced products or services that are delivered more quickly and are content with the current quality.

An organisation may become bureaucratic and apathetic, unable to make decisions for investment, and at risk of group think without the microfoundation of seizing. This is where an organisation is actively seeking new ideas and can put them into action; however, a lack of decision making may mean that this never occurs due to inertia. For example, an organisation might be well known for its technological, design and production processes and its ability to seek out opportunities for their use (reconfiguration and sensing), yet it may have an institutional reluctance to transition and invest in new technologies or settings. This may lead to slow development of change, leading to a long time-lag between the identification of a potential opportunity and the reconfiguration of routines to enable its exploitation.

### Implications

The dynamic capabilities view of improvement capability has important implications for researchers, healthcare managers and policy makers. This perspective suggests that there may not be an ideal combination of the microfoundations in all circumstances. A dynamic capabilities view does not support the operationalisation of improvement capability as a peformance metric in making summative judgements of the improvement capability of the organisation. Instead this perspective suggests that improvement capability as a series of microfoundations has different configurations contingent on organisational circumstances. This suggests that for some organisations, for example those in crisis, regulatory failure or financial disarray, the combination of microfoundations and associated dimensions of improvement capability will be different to the configuration in organisations that are heralded as best practice, outstanding and high performing. In the former, the microfoundation of sensing, with its emphasis on service-user focus, and stakeholders may be important to be able to re-build organisational confidence and capability to improve. In the latter, there may be more of a risk of organisational complacency and the microfoundation of seizing may be more important, with its emphasis on commitment and culture to help ensure continued development of improvement capability rather than potential decline and atrophy. Further research would be beneficial to understand the different configurations of microfoundations that may be needed in different circumstances, as this may help healthcare organisations to focus their attention on underdeveloped dimensions of improvement capability contingent on their circumstances, helping to ensure they can improve patient care more rapidly and reduce excessive performance variation.

Further research on the configuration of improvement capability is now required to examine how the dimensions in this framework inter-relate.

Furthermore, the implications of this framework are that “sensing” is important for researchers focussing on organisation context and there are already existing calls for more research on organisational context (Kaplan *et al.*, 2013; Øvretveit, 2011). Similarly, “seizing” is important for researchers who focus on the role of people within organisational settings, and empirical research points to the importance both leadership and employee commitment for the development of improvement capability across an organisation (Rother and Aulinger, 2017; Babich *et al.*, 2016; McGrath and Blike, 2015; Godfrey, 2013). When considering reconfiguration, there is a need to understand more about the specific routines to enact changes for improvement and to examine how reconfiguration works across differing sectors. Particularly, there is little empirical research in non-market situations, particularly from sectors outside of manufacturing and technology (Hogan *et al.*, 2011; Easterby-Smith *et al.*, 2009), including the public sector and healthcare.

Further research is also required to understand how the microfoundations and associated dimensions of improvement capability are orchestrated. The strength of this framework is that it views improvement capability as an interdependent bundle of routines that may lead to improved health system performance. The framework moves from a direct cause and effect understanding of each dimension to one which indicates more nuanced relationships in the configuration of the dimensions and has sought to explain why each microfoundation is important in the context of improvement capability and organisational performance. This explanation can strengthen the prospective assessment of healthcare organisations and help indicate the level, type and strength of development that organisations require to improve their performance across the dimensions. A more detailed understanding of the microfoundations of improvement capability is important for both employees, leaders, organisations and external bodies, such as regulatory agencies, as this may help to predict organisational performance trajectories, meaning that proactive measures could be taken before performance worsens due to inadequate improvement capability.

## Conclusions

The framework developed in this paper views improvement capability through the lens of the microfoundations of dynamic capabilities, conceiving it as a combination of eight dimensions which all play an important role in its formation and development. This paper has discussed the application of the dynamic capabilities view to analyse improvement capability and has contributed to a more nuanced understanding of its configuration. It has explored and explained how the dimensions of improvement capability interact as microfoundations of a dynamic capability and how this process of interaction may influence variations in organisational performance. This is important for policymakers and practitioners, in healthcare and other domains, who wish to improve performance, as it will help to identify and draw out specific areas of under or over-development of dimensions of improvement capability allowing resources and priorities to shift in response. The framework enables a more fine-grained understanding of improvement capability and its configuration and identifies avenues for further research.

This theoretically based framework provides a richer and more complete depiction of how improvement capability develops within an organisation. In addition, breaking improvement capability into its component parts enables managers and policy makers to identify the areas that are most in need of attention to support performance improvement.

## Figures and Tables

**Table I:** Improvement capability definitions

Definition	Author	Sector	Perspective
The ability to incrementally increase (manufacturing) performance using existing resources	Swink and Hegarty (1998)	Manufacturing	Process view
Improvement capability consists of organisational wide processes of focused and sustained incremental innovation	Bessant and Francis (1999)	Manufacturing	Process view
Resources and processes, supporting both the generation and the diffusion of appropriate innovations across the organisation	Adler *et al.* (2003)	Healthcare	Process view
It refers to the strength or proficiency of a bundle of interrelated organisational routines for incrementally improving existing products/processes	Peng *et al.* (2008)	Manufacturing	Process view
The ability to consistently improve current processes and learn new ones	Anand *et al.* (2009)	Cross-sector	Process view
The people that have the confidence, knowledge and skills to lead change	Bevan (2010)	Healthcare	Actor driven
Improvement capability is knowledgeable and skilled human resources able to lead the design of improvement initiatives, to achieve measurable results, execute (i.e. develop, test, measure and implement changes) the improvement efforts, and sustain the results	Kaminski *et al.* (2014)	Healthcare	Actor driven
An organisational strategy to implement and spread quality improvement programmes across their organisation	Babich *et al.* (2016)	Healthcare	Process view

**Table II:** Dimensions of improvement capability

Dimensions	Description
Organisational culture	Core values, attitudes and norms and underlying ideologies and assumptions within an organisation
Data and performance	Use of data and analysis methods to support improvement activity
Employee commitment	Encompasses the level of commitment and motivation of employees for improvement
Leadership commitment	Support by formal organisational leaders for improvement and performance
Process improvement and learning	Systematic methods and processes used within an organisation to make ongoing improvement through experimentation
Service-user focus	Identification and meeting of current and emergent needs and expectations from service users
Stakeholder and supplier focus	Extent of the relationships, integration and goal alignment between the organisation and stakeholders such as public interest groups, suppliers and regulatory agencies
Strategy and governance	Process in which organisational aims are implemented and managed through policies, plans and objectives

**Source:** Furnival *et al.* (2017)

**Table III:** Theory comparison

Theory	Resource-based view	Organisational ambidexterity	Dynamic capabilities view
Principles	Sustained superior performance depends on organisational strategic resources and strategic decision making. Resources must be valuable, imperfectly inimitable, rare and exploitable (VRIN) (Barney, 1995)	Defined as the ability of an organisation to simultaneously pursue both explorative (discontinuous) and exploitative (incremental) innovation (O’Reilly and Tushman, 2004)	Emphasises that superior and sustained performance depends on the organisational capacity to purposefully create, extend and modify its resource base through a bundle of organisational routines which must be sustained over time (Su *et al.*, 2014; Helfat *et al.*, 2007)
Primary focus	Resources	Knowledge	Routines
Components	Organisations need to structure, bundle and leverage organisational resources synchronously to achieve a superior performance (Sirmon *et al.*, 2007)	Organisations need to explore and exploit knowledge (Benner and Tushman, 2003; 2015)	Organisations need to sense, seize and reconfigure organisational routines (Teece, 2007)

**Table IV:** Improvement capability framework

Microfoundations	Microfoundation examples	Improvement capability dimension
Sensing	Customer needs Research and development Technology and industry developments	Service-user focus
		Stakeholder and supplier focus
Seizing	Culture Leadership Communication Developing business models Understanding markets and boundaries	Employee commitment
		Leadership commitment
		Organisational culture
Reconfiguring	Decentralisation Knowledge management Co-specialisation Governance	Data and performance
		Process improvement and learning
		Strategy and governance

**Sources:** Adapted from Teece (2007) and Furnival *et al.* (2017)
